# Facile synthesis of water-soluble luminescent mesoporous Tb(OH)_3_@SiO_2_ core-shell nanospheres

**DOI:** 10.1186/1556-276X-8-163

**Published:** 2013-04-10

**Authors:** Anees A Ansari, Joselito Labis, Abdullah S Aldwayyan, Mahmoud Hezam

**Affiliations:** 1 King Abdullah Institute for Nanotechnology, King Saud University, P.O. Box 2455, Riyadh, 11451, Saudi Arabia

**Keywords:** Luminescent mesoporous Tb(OH)_3_@SiO_2_ core-shell nanospheres, Optical properties, Photoluminescence

## Abstract

Luminescent mesoporous Tb(OH)_3_@SiO_2_ core-shell nanospheres were synthesized through W/O microemulsion process at ambient temperature. The negatively charged silica favors a coating of the positively charged Tb^3+^ composite. Thus, silicon layer was adsorbed on the surface of Tb(OH)_3_ groups to form Tb-O-Si through electrostatic interaction. X-ray diffraction, field emission transmission electron microscopy (FE-TEM), energy-dispersive X-ray spectrometry, and Fourier transform infrared, UV/Visible, and photoluminescence spectroscopies were applied to examine the phase purity, crystallinity, surface morphology, and optical properties of the core-shell nanospheres. The FE-TEM results have revealed typically ordered mesoporous characteristics of the material with monodisperse spherical morphology in a narrow size distribution. The luminescent mesoporous core-shell nanospheres exposed remarkable splitting with broadening in the emission transition ^5^D_4_ → ^7^F_5_ (543 nm). In addition, the luminescent mesoporous core-shell nanospheres emit strong green fluorescence (from Tb^3+^) in the middle of the visible region under 325 nm (3.8) excitation. The luminescent mesoporous Tb(OH)_3_@SiO_2_ core-shell nanospheres can therefore be exploited as fluorescent probes in biomarkers or biolabeling, optical sensing, and drug delivery system. Further, these nanospheres could have potential use as scattering layers in dye-sensitized solar cells.

## Background

During the past decade, great efforts have been devoted to the preparation of mesoporous core-shell nanomaterials due to their potential applications in drug-delivery carriers [1-3], optical bioprobes [4], biomarkers [5], and fluorescent biolabeling [6,7]. These mesoporous core-shell nanomaterials possess attractive features such as well-defined and controllable pore size, high pore volume, large surface area, non-toxic nature, easily modified surface properties, and good biocompatibility [8]. However, the use of bulk mesoporous silica in many applications suffers from many limitations, especially in the targeted drug delivery mechanisms as carrier and drug kinetics marker in the pharmacological research [9,10]. Recently, luminescent metal-doped mesoporous materials, which can be tracked or monitored to evaluate the efficiency of the drug release, have become a research hotspot [1-3,11-14]. The integration of luminescent metal-doped nanocrystals with mesoporous silica to form core-shell structures is undoubtedly of great value because mesoporous shells not only offer high surface area for derivation of numerous functional groups but also provide accessible large pore channels for the adsorption and encapsulation of biomolecules and even functional nanoparticles. Up to date, a lot of techniques have been reported for the synthesis of luminescent metal-doped mesoporous silica core-shell structures, such as mesoporous silica encapsulating quantum dots/nanoparticles [15,16], luminescent metal nanoparticles [17], and luminescent lanthanide metal nanoparticles [18,19]. However, all core particles are spherical. Among various luminescent metal ion-doped mesoporous core-shell nanoparticles, luminescent lanthanide-doped core-shell nanoparticles are promising because of their good chemical durability, thermal stability, and optical features. Moreover, such luminescent Ln^3+^-doped mesoporous core-shell nanoparticles have sharp emission lines, long lifetimes, superior photostablility, large Stokes shifts, good chemical/physical stability, and low toxicity [8]. At present, there are only a few reports on the synthesis of luminescent lanthanide-doped mesoporous core-shell nanospheres. For example, Qian et al. have synthesized mesoporous-silica-coated upconversion fluorescent nanoparticles through water/oil (W/O) microemulsion process for photodynamic therapy [11]. Yang et al. prepared mesoporous silica encapsulating upconversion luminescence rare-earth fluoride nanorods by using the surfactant-assisted sol-gel process [18]. Lin and his coworkers have been synthesizing mesoporous upconversion luminescent NaYF_4_:Yb^3+^/Er^3+^@nSiO_2_@mSiO_2_-doped core-shell nanospheres via a simple two-step sol-gel process [1]. Although it is well accepted that uniform spherical core-shell nanoparticles with lower surface defects are preferred to improve optical properties, little effort has been devoted to the synthesis of mesoporous core-shell nanospheres. However, in most of these mentioned approaches, the synthesis process of the core-shell nanoparticles involves a multistep high-temperature preparation and less biocompatibility, such as first preparation of core (seed spherical nanoparticles) and then coating a shell of silica on the surface of the nanoparticles. Therefore, it is desirable to develop a facile, low-cost, and large-scale approach to prepare water-soluble, luminescent, mesoporous core-shell and well-dispersed spherical nanoparticles. To the best of our knowledge, the luminescent lanthanide mesoporous core-shell nanospheres have been rarely fabricated. In the present work, a method for direct coating of β-diketonate stabilized the luminescent metal-chelating complex with silica shells by a seeded polymerization technique is proposed. The method does not require any coupling molecules and is based on a method that our group has used for the preparation of monodispersed mesoporous silica core-shell nanospheres [8,20]. The concentrations of water, ammonia, luminescent metal-chelating complex, cetyltrimethyl-ammonium bromide (CTAB), and silicon alkoxide are important factors governing particle size and distribution in microemulsion reaction of alkoxides. Fine control of the amount of silicon alkoxide, ethanol, water, and ammonia (catalyst) is used to prevent secondary silica nucleus formation and to provide rapid shell growth.

Herein, we report a facile synthesis of water-soluble, luminescent Tb^3+^-doped mesoporous core-shell nanospheres via a modified W/O microemulsion process. We are employing Tb(acac)_3_·3H_2_O as doping chelating complex in the silica framework which shows green luminescence in visible region. In addition, the size of the nanospheres could be fine-tuned from 10 to 130 nm, which is very crucial for applications in the biofield.

## Experimental

### Materials and methods

Terbium oxide (99.99%, Alfa Aesar, Karlsruhe, Germany), tetraethyl orthosilicate (TEOS, 99 wt.% analytical reagent A.R.), Cyclohexane (BDH, England, UK), C_2_H_5_OH, HNO_3_, NH_4_OH, n-hexanol, and Triton X-100 (Sigma-Aldrich, St. Louis, MO, USA) were used as starting materials without any further purification. Tb(NO_3_)_3_·6H_2_O were prepared by dissolving the corresponding oxides in diluted nitric acid, and nanopure water was used for preparation of solutions. Ultrapure deionized water was prepared using a Milli-Q system (Millipore, Bedford, MA, USA). All other chemicals used were of reagent grade.

### One-pot synthesis of luminescent mesoporous Tb(OH)_3_@SiO_2_ core-shell nanospheres

Luminescent mesoporous Tb(OH)_3_@SiO_2_ core-shell nanospheres were prepared via a modified W/O microemulsion process as follows: before the nanoparticle preparation, the Tb(acac)_3_·3H_2_O chelating complex was prepared by a reported method [21]. In a typical procedure, firstly, microemulsion was prepared by mixing 3.54 ml of Triton X-100, 15 ml of cyclohexane, and 4.54 ml of n-hexanol under constant stirring at room temperature. Then, 2 ml of an aqueous solution of Tb(acac)_3_·3H_2_O chelating complex (1 M) was added into the mixture. After that, a mixed solution containing TEOS (2 ml), H_2_O (5 ml), and CTAB (50 mg) was added. In the presence of TEOS, a polymerization reaction was initiated by adding 1 ml of NH_4_OH. The resulting reaction was allowed to continue for 24 h. After the reaction was completed, the luminescent mesoporous nanospheres were isolated by acetone followed by centrifuging and washing with ethanol and water several times to remove any surfactant molecules.

### Characterization

The X-ray diffraction (XRD) of the powder samples was examined at room temperature with the use of PANalytical X’Pert X-ray diffractometer (Almelo, The Netherlands) equipped with a Ni filter using Cu K_α_ (*λ* = 1.54056 Å) radiations as X-ray source. The size and morphology of the samples were inspected using a field emission transmission electron microscope (FE-TEM) equipped with an energy-dispersive X-ray spectrometer (EDX) (FE-TEM, JEM-2100F, JEOL, Akishima-shi, Japan) by operating at an accelerating voltage of 200 kV. EDX analysis was used to confirm the presence of the species. Samples for TEM were prepared by depositing a drop of a colloidal ethanol solution of the powder sample onto a carbon-coated copper grid. The FTIR spectra were recorded using a PerkinElmer 580B IR spectrometer (Waltham, MA, USA) using the KBr pellet technique in the range of 4,000 to 400 cm^-1^. The UV/vis absorption spectra were measured using a PerkinElmer Lambda-40 spectrophotometer, with the sample contained in a 1-cm^3^ stopper quartz cell of a 1-cm path length, in the range of 190 to 600 nm. Photoluminescence spectra were recorded on Horiba Synapse 1024x 256 pixels, size of the pixel 26 microns, detection range: 300 (efficiency 30%) to 1000 nm (efficiency: 35%) (Kyoto, Japan). In all experiments, a slit width of 100 microns is employed, ensuring a spectral resolution better than 1 cm^-1^. All measurements were performed at room temperature.

## Results and discussion

**Figure 1:**
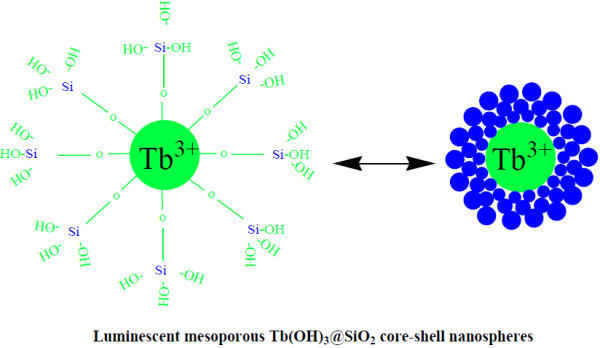
**Schematic diagram of the synthesis process of luminescent mesoporous Tb(OH)**_**3**_**@SiO**_**2**_**core-shell nanospheres.**

The synthesis of the luminescent mesoporous core-shell structured Tb(OH)_3_@SiO_2_ nanospheres is presented in Figure 1. Typically, the as-prepared luminescent Tb(OH)_3_@SiO_2_ nanospheres were treated by a modified W/O microemulsion procedure to result in the formation of the silica-Tb(OH)_3_ composites with a non-porous silica layer (denoted as Tb(OH)_3_@SiO_2_). Subsequently, CTAB was selected as the organic template for the formation of the outer mesoporous silica layer on Tb(OH)_3_@SiO_2_. The detailed experimental processes were previously presented in the ‘Experimental’ section.

**Figure 2:**
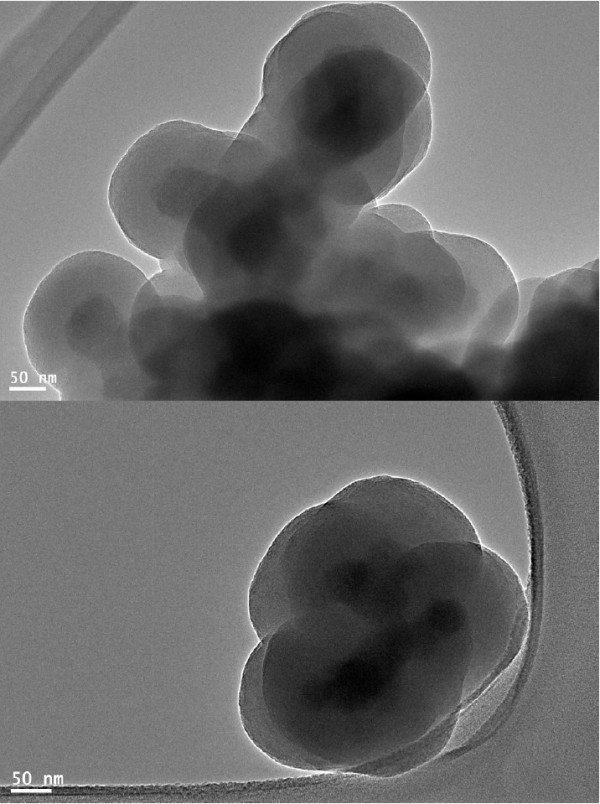
**Typical FE-TEM micrographs of luminescent mesoporous Tb(OH)**_**3**_**@SiO**_**2**_**core-shell nanosphere.**

The representative FE-TEM micrographs of the luminescent mesoporous silica-coated Tb(OH)_3_ nanospheres, with (a) an inset of the mesoporous core-shell part, and (b) at a high magnification of the outer layer are displayed in Figure 2. TEM micrograph in Figure 2a shows that the nanospheres are aggregated, mesoporous, spherically shaped, and well-distributed to some extent. The size of the nanospheres is between 120 and 140 nm. Mesoporous pore sizes along with small particle sizes (*<*150 nm) are advantageous and favorable for drug delivery applications. It can be seen that the deposition of silica layer has little influence on the morphologies of the Tb(OH)_3_ nanospheres. As observed in Figure 2, the deposition of silica layer on the surface of nanospheres has increased the morphologies of their parent nanospheres by around 40 to 50 nm. Although this TEM sample exhibits overlapped silica-coated Tb(OH)_3_, the contrast between the light-gray amorphous silica layer (50-nm thick) and the dark Tb(OH)_3_ layer (approximately 50 nm in diameter) is apparent.

**Figure 3:**
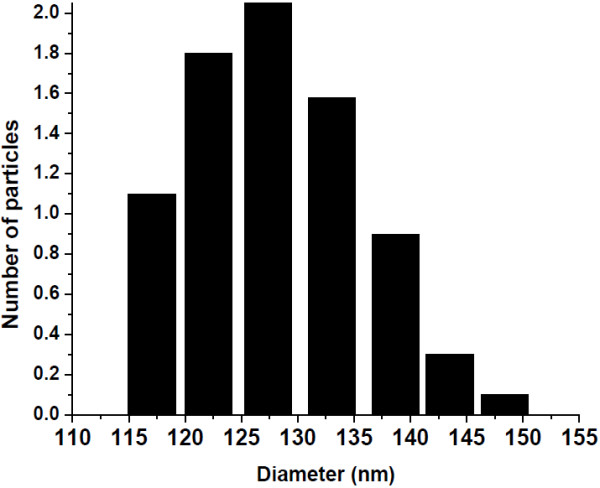
**Size distribution for the luminescent mesoporous Tb(OH)**_**3**_**@SiO**_**2**_**core-shell nanosphere in ethanol deduced from dynamic light-scattering experiments.**

Silica-surface-modified core-shell nanospheres reveal high solubility in water accompanied by a strong decrease of their solubility in organic solvents. As evident from dynamic light scattering (DLS) measurement, the core-shell nanospheres are not very well separated (aggregated) in this solvent (ethanol). The DLS measurements indicate the average hydrodynamic diameter of the core-shell nanospheres in ethanol about 120 to 140 nm (Figure 3). This size distribution is well in accord with the mean particle size observed in the FE-TEM micrographs. As evident from the literature, broad size distribution of nanoparticles derived from TEM images and DLS studies is ideal for bio-tagging experiments; because of bio-tagging, experiments will always be performed in solution.

**Figure 4:**
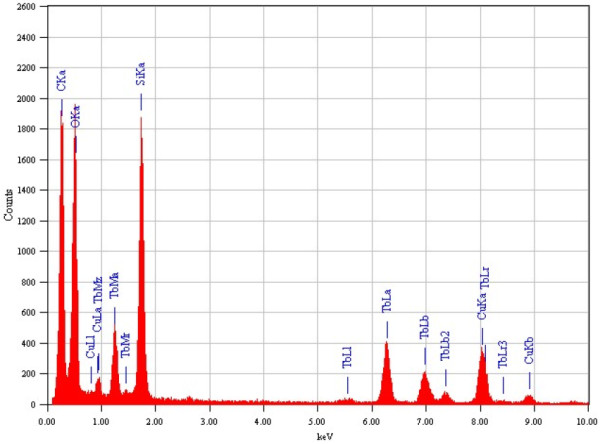
**EDX image of the luminescent mesoporous Tb(OH)**_**3**_**@SiO**_**2**_**core-shell nanosphere.**

The EDX analysis was performed to confirm the chemical stoichiometry and the successful doping of terbium ion in the silica core-shell nanospheres. The EDX analysis of nanospheres provides an additional evidence of the synthesis luminescent mesoporous silica-coated terbium hydroxide core-shell nanospheres. From Figure 4, the strongest Si peaks are clearly indicated together with Tb and O peaks. It should be noted that the origin of strong Cu peaks that appeared in the EDX spectra are from the copper micrometer grids. The C peak also came from the carbon-coated Cu-TEM grid. No other impurities are evident in the figure, implying that the resulting Tb(OH)_3_@SiO_2_ nanospheres are pure in chemical composition.

**Figure 5:**
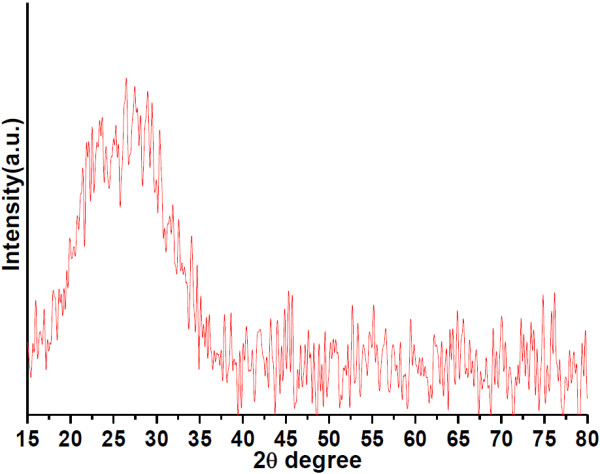
**Wide-angle X-ray diffraction pattern of luminescent mesoporous Tb(OH)**_**3**_**@SiO**_**2**_**core-shell nanosphere.**

The X-ray diffraction pattern of the luminescent mesoporous core-shell nanoparticles prepared by W/O microemulsion system is shown in Figure 5. The XRD result shows that the nanoparticles have only a broad peak located at 15° to 35° spectrum, and no sharp diffraction peak corresponding to the crystalline structure. There are no detectable diffractions attributed to the Tb^3+^ ions crystalline phase. The broad peak is attributed to the existence of amorphous silica (JCPDS no. 29-0085) components or to ultra-small crystalline materials where diffraction peaks cannot be well resolved [3,22]. Therefore, it was found that the luminescent functionalized (Tb^3+^) in the silica framework expanded the nanopores and rearranged the Si-O-Si network structures without any impurities. This result is similar to that for reported silica-coated iron oxide nanoparticles and shows that the Tb chelate-doped silica nanoparticles are non-crystalline materials. And the Tb chelate molecules in the nanoparticles exist in a noncrystalline or ultra-small crystalline state [19,22-24].

**Figure 6:**
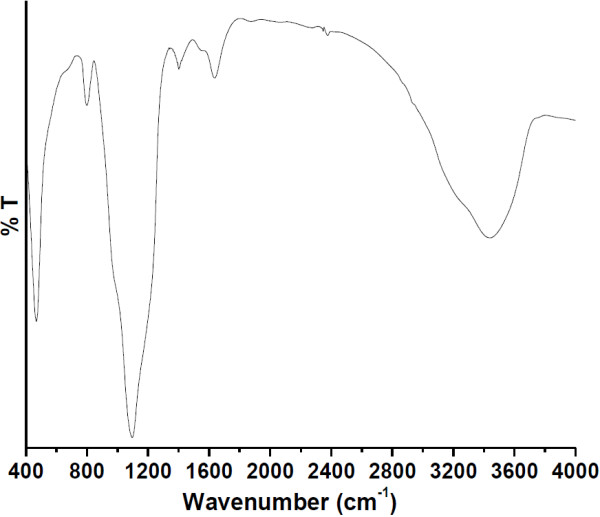
**FTIR spectrum of the prepared luminescent mesoporous Tb(OH)**_**3**_**@SiO**_**2**_**core-shell nanosphere.**

FTIR spectroscopy was performed to confirm the synthesis of luminescent mesoporous Tb(OH)_3_@SiO_2_ nanoparticles. As illustrated in Figure 6, a wide band at around 2,900 to 3,750 cm^-1^ is observed which is attributed due to the presence of O-H and Si-OH groups on the surface of nanospheres. Two weak-intensity infrared bands measured in the middle of infrared region located at 1,365 and 1,639 cm^-1^ are due to the bending vibrations of the hydroxyl groups (-OH), which are associated on the surface of nanospheres. The spectrum exhibited strong infrared absorption bands around 1,090 cm^-1^ which originate from the Si-O-Si asymmetric and symmetric stretching [8,20]. The band at around 792 cm^-1^ is assigned to the Si-OH stretching. An intense sharp band at 473 cm^-1^ is attributed to the Tb-O-Si stretching vibrational mode. Furthermore, the intensity and broadening of the bands indicated a large number of OH groups and Si-OH molecules present on the surface. This could play an important role including biocompatibility in biological systems, functionality, and high colloidal stability under different conditions [24]. These results corroborate with the analysis of FE-TEM micrographs, EDX, and XRD analysis which confirmed that silica had been successfully encapsulated on the surface of Tb(OH)_3_ molecules.

### Optical properties

**Figure 7:**
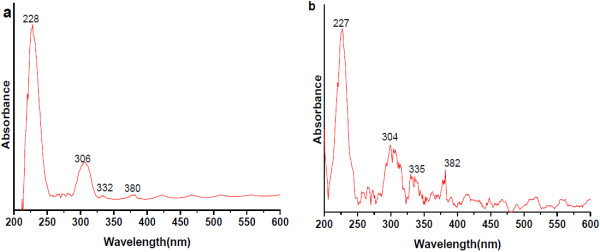
**UV/vis absorption spectra of luminescent mesoporous Tb(OH)**_**3**_**@SiO**_**2**_**core-shell nanosphere suspended in (a) ethanol and (b) deionized water.**

Figure 7 illustrates the optical absorption spectra of the as-synthesized luminescent mesoporous Tb(OH)_3_@SiO_2_ core-shell nanospheres. As shown in Figure 7, the absorption spectra were measured in ethanol and deionized water in similar concentrations. The absorption spectra in ethanol displayed an intense band located at 228 nm with a middle intensity band around 306 nm. The absorption at 228 nm originates from the silica parts, which agrees with the spectra of previous observations [25-28], and the middle intensity absorption band at 308 nm likely originates from the terbium hydroxide [26-28]. The spectrum displayed some small intensity absorption transitions in visible region which correspond to the forbidden 4f^8^-4f^7^5d transitions of Tb^3+^ ion usually weak in silica matrices. These prominent levels of terbium ions observed are assigned to the appropriate electronic transitions as ^7^F_6_ → ^5^G_4_ (304 nm), ^7^F_6_ → ^5^L_10_ (335 nm), and ^7^F_6_ → ^5^G_6_ (382 nm) [26-28]. The absorption spectrum confirms the formation of Tb(OH)_3_ nanoparticles along with silica surface in the core-shell nanospheres [27]. The addition of silica layer is marked by a pronounced scattering and sharpening of the absorption peak, and weak terbium hydroxide absorption transitions are appearing in the Tb(OH)_3_@SiO_2_ colloid. Obviously, the silica-surface-modified terbium hydroxide nanoparticles is screened by the strong scattering from the silica colloid. These results can be corroborated visually by the loss of the characteristic light-yellow color to a dirty-white-colored solution with fine colloidal dispersion after silica adsorption on the terbium hydroxide surface. This observation is indicative of colloidal stability which is essential for further biological targeting. We observed similar trend in the absorption spectra measured in deionized water as seen in Figure 7b.

**Figure 8:**
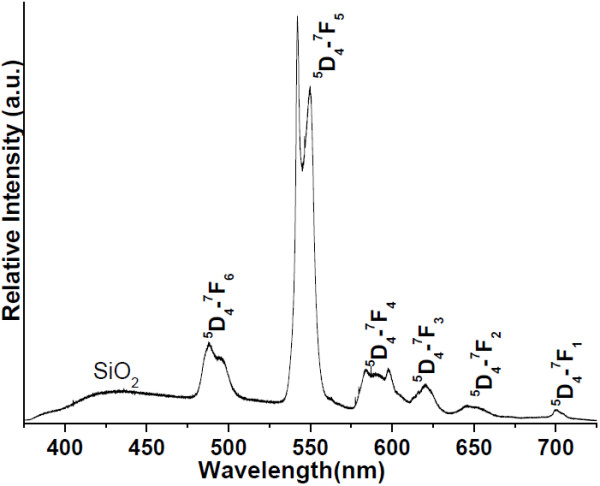
**Photoluminescence spectrum of luminescent mesoporous Tb(OH)**_**3**_**@SiO**_**2**_**core-shell nanospheres.**

Figure 8 presents the photoluminescence properties of the luminescent mesoporous Tb(OH)_3_@SiO_2_ core-shell nanospheres under the excitation of 325 nm (3.82 eV) and recorded by fluorescence spectrometer at room temperature. As displayed in Figure 8, the emission spectrum reveals six strong transitions in the visible region and can be observed at 490 nm (2.53 eV; ^5^D_4_ → ^7^F_6_), 543 nm (2.28 eV; ^5^D_4_ → ^7^F_5_), 590 nm (2.10 eV; ^5^D_4_ → ^7^F_4_), 613 nm (2.00 eV; ^5^D_4_ → ^7^F_3_), 654 nm (1.90 eV; ^5^D_4_ → ^7^F_2_), and 700 nm (1.76 eV; ^5^D_4_ → ^7^F_0_), with the most prominent hypersensitive ^5^D_4_ → ^7^F_5_ transition located in the range of 534 to 560 nm, corresponding to the green emission, in good accordance with the Judd–Ofelt theory [29-31]. A broad band between 370 and 475 nm is also observed which is caused by the silica emission. The luminescent mesoporous core-shell spectrum produced very typical band features of ^5^D_4_ → ^7^F_6_, ^5^D_4_ → ^7^F_5_, and ^5^D_4_ → ^7^F_4_ transitions in the wavelength region 478 to 506, 533 to 562, and 575 to 608 nm, respectively. Among emission transitions ^5^D_4_ → ^7^F_5_ (543 nm) was most influenced and exhibits the hypersensitivity in the spectrum. Here we observe that the emission intensity of Tb^3+^ is significantly dependent on the amount of silica core-shell network. The possible explanation is that Tb^3+^ doped into the network of SiO_2_ would produce non-bridging oxygen, which paved the way for the broadening of 4f^8^ → 4f^7^5d transition band for the co-doped sample. By exciting at this wavelength, the emission intensity of the co-doped sample is markedly increased compared to the Tb^3+^ alone doped sample.

The figure shows significant differences in the band shapes of the emission transitions such as ^5^D_4_ → ^7^F_6_, ^5^D_4_ → ^7^F_4_, and ^5^D_4_ → ^7^F_3_, and this is attributed to the differences in their structure and interaction of Si molecules with the 4f-electrons of the metal ions. These intensity enhancement effects may be related to the change in the strength and symmetry of the crystal field produced by the silica network [32]. The broadening and splitting of spectral lines are also observed and are induced by the change in chemical environment of Tb^3+^ ions during the formation of new chemical bonds between silica network and terbium hydroxide. The luminescence spectrum displayed well-defined crystal-field splitting of the narrow luminescence lines, which are induced by the change in chemical environment of Tb^3+^ ions during the formation of new chemical bonds between silica network and terbium hydroxide. This shows that the crystal field is very similar for most Tb^3+^ ions as previously reported in the literature [32-34]. This is expected because the Tb^3+^surface sites are converted into volume sites by growing the silica core-shell, thereby reducing the number of different Tb^3+^ sites in the material. The highest branching ratio corresponds to the ^5^D_4_ → ^7^F_5_ transition (543 nm), and this transition may therefore be considered to be a possible laser transition.

## Conclusions

In summary, luminescent mesoporous silica-coated terbium hydroxide core-shell nanospheres were synthesized through W/O microemulsion process. The FE-TEM, EDX, XRD, and FTIR techniques were used to characterize the morphology and composition of the core-shell nanospheres. The optical spectra of the core-shell nanospheres confirmed that the properties of the terbium ion were strongly affected by the doping procedure. The emission spectrum of Tb(OH)_3_@SiO_2_ nanospheres shows the characteristic emission peaks of Tb^3+^ and a weak background band of SiO_2_.

The luminescent intensity of the hypersensitive transition (^5^D_4_ → ^7^F_5_) in core-shell nanospheres is greatly enhanced because the non-radiative processes at or near the surface of the nanospheres is greatly reduced. The strong green emission of Tb^3+^ in core-shell nanospheres results from an efficient energy transfer from silica to Tb^3+^, in which the non-bridging oxygen atom is present between the metal ion and silica frameworks. The luminescent metal ion inside the nanospheres has two functional entities which allow optimizing their luminescence and aqueous solubility separately. The study of these novel composite nanospheres is of profound importance for the new applications in biomarkers and drug delivery, as well as in nucleic acid assay. The luminescent property of these materials as well as their reported light upconversion can have a potential use in dye-sensitized solar cells as a scattering layer for better harvesting of solar light, which will be subject for future investigation.

## Competing interests

The authors declare that they have no competing interests.

## Authors’ contributions

AAA carried out the synthesis of the water-soluble luminescent mesoporous Tb(OH)_3_@SiO_2_ core-shell nanospheres, participated in the characterizations, and drafted the manuscript. JL carried out the characterizations of the nanospheres and participated in the editing of the manuscript. ASA and MH participated in the discussion and design of the study for the possible application of the nanospheres in solar-cells. All authors read and approved the final manuscript.
